# Off-Line Evaluation of Mobile-Centric Indoor Positioning Systems: The Experiences from the 2017 IPIN Competition

**DOI:** 10.3390/s18020487

**Published:** 2018-02-06

**Authors:** Joaquín Torres-Sospedra, Antonio R. Jiménez, Adriano Moreira, Tomás Lungenstrass, Wei-Chung Lu, Stefan Knauth, Germán Martín Mendoza-Silva, Fernando Seco, Antoni Pérez-Navarro, Maria João Nicolau, António Costa, Filipe Meneses, Joaquín Farina, Juan Pablo Morales, Wen-Chen Lu, Ho-Ti Cheng, Shi-Shen Yang, Shih-Hau Fang, Ying-Ren Chien, Yu Tsao

**Affiliations:** 1 Institute of New Imaging Technologies, Universitat Jaume I, 12071 Castelló, Spain; gmendoza@uji.es; 2 Centre for Automation and Robotics (CAR), CSIC-UPM, 28500 Arganda del Rey, Spain; fernando.seco@car.upm-csic.es; 3 Algoritmi Research Centre, University of Minho, 4800-058 Guimarães, Portugal; maria.j.nicolau@algoritmi.uminho.pt (M.J.N.); antonio.costa@algoritmi.uminho.pt (A.C.); 4 AraraDS, Monseñor Sótero Sanz 161, 8340457 Santiago, Chile; joaquin@ararads.com (J.F.); juanpablo@ararads.com (J.P.M.); 5 Department of Electrical Engineering, Yuan Ze University, Zhongli 32003, Taiwan; dennis70413@gmail.com (W.-C.L.); s1054605@mail.yzu.edu.tw (W.-C.L.); s1054601@mail.yzu.edu.tw (H.-T.C.); 6 Faculty for Geomatics, Computer Science and Mathematics, HFT Stuttgart—University of Applied Sciences, 70174 Stuttgart, Germany; 7 Estudis d’Informàtica, Multimèdia i Telecomunicació, Universitat Oberta de Catalunya, Rambla del Poblenou 156, 08018 Barcelona, Spain; aperezn@uoc.edu; 8 Internet Interdisciplinary Institute IN3, Av. Carl Friedrich Gauss 5, 08860 Castelldefels, Barcelona, Spain; 9 Centro de Computação Gráfica (CCG), 4800-058 Guimarães, Portugal; filipe.meneses@ccg.pt; 10 Department of Electrical Engineering, National Ilan University, Yilan 26047, Taiwan; shen49853@gmail.com; 11 Research Center for Information Technology Innovation, Academia Sinica, Taipei 11529, Taiwan; yu.tsao@citi.sinica.edu.tw

**Keywords:** indoor positioning and navigation, Wi-Fi fingerprinting, sensor fusion, competitions, benchmarking

## Abstract

The development of indoor positioning solutions using smartphones is a growing activity with an enormous potential for everyday life and professional applications. The research activities on this topic concentrate on the development of new positioning solutions that are tested in specific environments under their own evaluation metrics. To explore the real positioning quality of smartphone-based solutions and their capabilities for seamlessly adapting to different scenarios, it is needed to find fair evaluation frameworks. The design of competitions using extensive pre-recorded datasets is a valid way to generate open data for comparing the different solutions created by research teams. In this paper, we discuss the details of the 2017 IPIN indoor localization competition, the different datasets created, the teams participating in the event, and the results they obtained. We compare these results with other competition-based approaches (Microsoft and Perf-loc) and on-line evaluation web sites. The lessons learned by organising these competitions and the benefits for the community are addressed along the paper. Our analysis paves the way for future developments on the standardization of evaluations and for creating a widely-adopted benchmark strategy for researchers and companies in the field.

## 1. Introduction

Indoor positioning, location and navigation are hot research topics whose market is expected to growth at a 42% annual rate in the next five years, with an expected worth of 41 billion USD by 2022 and 58 billion USD by 2023 [1,2]. The keys for the success of these areas are diverse: the variety of devices that can be used for localization and tracking (smartdevices [3,4], and open hardware platforms with commercial off-the-shelf or custom accessories [5,6,7], among others); the target applications ( automated stocktaking in a warehouse [8], ambient assisted living [9], vehicle navigation [10], urban mobility [11], health and patient’s monitoring [12,13,14], robotics [15], epidemic tracking [16], customer tracking [17], finding objects [18], among many others); and the background diversity of the involved professionals, which includes data scientists, machine learning experts, computer scientists, telecommunications and industrial engineers and physicists, among many others.

In contrast to outdoor positioning, where the majority of systems and applications rely on GNSS, many technologies have emerged to provide positioning indoors. They are based on: Wireless Communication Technologies (Wi-Fi [5,19,20], BLE [18,21], RFID [22,23], FM [24], Ultra Wide Band [18,25,26]), Ultrasounds [27,28], IMU’s [13,29,30,31]; signals of opportunity [32], Optical and Vision [11], visible light [4], and Magnetic, [33] among others. Selecting the right technology might not be an easy task since many features have to be balanced, such as target application, deployment costs, required accuracy, tolerable uncertainty or needed computational resources. In general, each base indoor positioning technology has a well-defined domain of applications: Wi-Fi fingerprinting is usually applied in smartphone applications, whereas UWB is more suitable for complex applications where higher accuracy is required. Hybrid systems which combine several technologies in a single system for enhanced location accuracy or robustness also exist ([11,34,35,36]).

The topics related to indoor positioning are very diverse, and they are enriched by the expertise of the community working on it. The International Conference on Indoor Positioning and Indoor Navigation (IPIN) [37] is the venue where researchers, professionals, and companies (featuring diverse backgrounds and interests) annually meet to debate about technologies, deployments and practical applications of indoor positioning. However, the works presented in this conference, and other related conferences and publications, tend to show the results of experiments carried out in the authors’ facilities using their own testbeds. The diversity on evaluation approaches makes it impossible to compare two solutions with the information provided about their respective works, since the evaluation set-up (scenario, contexts, suppositions, strategies, and so on) might be radically different in both set-ups. In addition, researchers are not able to reproduce the evaluation set-ups found in the literature if the collected data are not made available by the researchers.

An illustrative example of the evaluation diversity problem can be found in two works presented at the 2017 IPIN conference [38,39]. The former shows the evaluation results in some corridors (inside an area of ≈30m×90m, with 90 reference points), whereas the latter shows results in an office (11.5 × 28 m${}^{2}$, 5 APs and 1645 calibration fingerprints), a trade fair (3422 m^2^ and 18 APs) and a warehouse (826.32 m^2^ and 70 Bluetooth beacons). Although both works present valid results, the differences in covered area, density and distribution of APs, number of reference fingerprints, and so on, make quantitative comparisons among them difficult.

Since 2015, the IPIN Conference holds an indoor positioning competition [40]. Through the definition of different tracks, the competition aims to provide an unbiased comparison of the solutions provided by the competitors in different contexts. This paper describes the results from the latest edition of the IPIN competition, held in Sapporo, Japan (October 2017), focusing especially in the Smartphone-based (off-site) track. This track (Track 3 in the competition) allowed the off-site evaluation of working indoor positioning systems that rely on data collected using smartphones. The competition organizers provided pre-collected databases for training, validation and evaluation to competitors. The use of databases allows an evaluation of the competing solutions that is independent of how data are collected, since all users have exactly the same data, i.e., competitors cannot deploy infrastructure and perform custom calibration mapping that would affect the system evaluation. Despite our focus on Track 3, the authors of this paper believe that our conclusions are applicable to the IPIN competition as a whole, since there is a common need to overcome the diversity evaluation problem previously mentioned and provide useful tools and frameworks for evaluating indoor positioning systems.

Nowadays, providing databases with pre-recorded data is becoming more popular in the scientific literature, since it provides a way for third-party researchers to validate the positioning method proposed by the authors or provide alternative methods. In our opinion, sharing positioning data fosters the development of new localization algorithms.

Databases can be introduced by publishing a data descriptor paper (e.g., [24,33,41]) or by providing supplementary data in a full research article (e.g., [8]). The 2017 IPIN conference organized the first session dedicated to evaluation (session chaired by Nobuhiko Nishio on 19 September 2017 with five contributions), where databases and other evaluation aspects were the main goal of the works. In previous editions, the evaluation topics were considered residual and presented under the umbrella of other main research works.

The creation of the ISC (International Standards Committee) of IPIN [42] was announced during the 2017 IPIN Conference. This Committee aims to develop and promote open standards for indoor positioning and indoor navigation through the collaboration of academia, industry and government organizations around the globe, where evaluation will also be a fundamental pillar.

The competition organization, the dedicated sessions and the proliferation of standards, such as ISO/IEC 18305:2016 [43], demonstrate that the indoor community is interested in providing databases and harmonize the evaluation of indoor positioning systems under equal conditions. This paper presents our experiences through the 2017 IPIN competition in joining together normalized database creation, experimentation, evaluation metrics and a discussion about comparing indoor positioning systems.

To sum up, the main aim of this paper is threefold: showing the results of the competition, demonstrating that all competitions are useful for the research community and discussing about improving the evaluation of indoor positioning systems.

The rest of this paper is organized as follows: Section 2 introduces the evaluation problems found in the literature and the initiatives to evaluate indoor positioning systems. Section 3 describes the IPIN competition, with special interest of the Smartphone-based (off-site) track and competing systems. Section 4 presents the competition results. Section 5 shows the lessons learned, from the organization’s and the participants’ points of view.

## 2. Related Work

The indoor positioning community is concerned about the evaluation problem, as demonstrated by the proliferation of many different initiatives to promote the evaluation of indoor positioning systems.

Several specific initiatives for evaluating indoor positioning systems have been proposed. First, operative indoor positioning prototypes are being evaluated through competitions, which might include tracks to cover different contexts. Those competitions not only evaluate working systems, but, in some on-line cases, also the ability for deploying a proper infrastructure in a short period of time. Second, open evaluation benchmarking testbeds and web platforms are emerging to provide a large variety of evaluation setups for indoor positioning systems. Third, many researchers are providing the collected data used in their experiments in public/general-purpose repositories or in personal/institutional websites.

In general, the indoor community is adopting more open and transparent evaluation procedures which enable further comprehensive comparative works. The adoption of open evaluation approaches should be seen as a unique opportunity to share different complementary ideas, increase the motivation of the indoor community to improve the accuracy of current systems and, above all, consolidate the indoor positioning as an emerging topic. Since the indoor positioning topics are diverse, they are expected to have multiple open alternatives for open evaluation.

### 2.1. Indoor Competitions

The Microsoft Indoor Localization Competition at the International Conference on Information Processing in Sensor Networks (IPSN) is one of the most popular indoor localization competitions [44,45,46]. In all editions, teams from industry and academia were encouraged to participate in an event where their performance was compared in the same space in the conference venue. Although all positioning techniques were allowed a priori, some restrictions were applied: end-users’ manual measurements were forbidden, competitors could deploy up to 10 devices as infrastructure to provide indoor positioning (not available for other competitors), jamming other deployments was not allowed, and the competitors had to show their results on an easy-to-carry portable device (smartphone, tablet, laptop or similar).

The EvAAL initiative [47,48,49] organized the first public international (indoor) localization competitions in 2011–2013, which were focused on indoor localization and tracking for ambient assisted living (AAL). The 2012 and 2013 editions included additional tracks, such as the activity recognition for AAL. Later, the International Conference on Indoor Positioning and Indoor Navigation carried out its first competition in 2014 by adopting the EvAAL framework. In 2015, the EvAAL-ETRI competition was held in the IPIN conference [50]. The IPIN Competition has formally adopted the EvAAL framework since 2016 [51].

PerfLoc [52] is an on-going prize competition organized by the US National Institute of Standards and Technology (NIST) for development and performance evaluation of smartphone indoor localization and tracking applications based on a huge dataset [52]. The provided data cover smartphone sensors, Radio Frequency (RF) signal strength, and GPS data collected in four large buildings by means of four different smartphones. The generated dataset covers more than 30,000 square meters of space, more than 900 evaluation points professionally surveyed and a total of ≈16 h of recorded data with each smartphone.

The Geo IoT World conference also provided an indoor location testbed [53] where some leading solutions in indoor positioning were assessed in 2016 and 2017. The testbed admits different positioning technologies: the phone-based segment, the infrastructure-free segment and solutions with a dedicated hardware. The two editions of the Indoor Location Testbed evaluated 12 solutions from 9 companies, such as: BlooLoc, GipsTech, Accuware (former Navizon), HERE, Indoo.rs, Lambda4, Movin, NexToMe or Senion.

### 2.2. On-Line Benchmarking Platforms

In addition to the competitions, platforms allowing the comparison of different solutions have great importance.

The EVARILOS benchmarking platform (EBP) [54], with a competition held in 2014, had the objective of automating the evaluation process [55]. The EVARILOS benchmarking proposal defines an evaluation criterion [56] which is aligned with the upcoming ISO/IEC JTC 1/SC 31 standard for evaluating RF-based IPSs. Although it targets radio frequency-based systems, some of their ideas could be applied to broader scenarios. In fact, the EBP has been useful for objectively capturing the performance and comparing several solutions using multiple evaluation metrics in multiple events and scenarios [45,57].

The IndoorLoc Platform [58] is a public repository for comparing and evaluating indoor positioning algorithms. The platform is a centralized website where researchers can access a public repository of datasets for indoor positioning, evaluate their indoor positioning system on well-established reference databases with blind test sets, analyze positioning methods and interact with the platform.

### 2.3. Databases

Although the IndoorLoc Platform facilitates the access to reference databases, there are other databases available online for download.

It has become a trend to publish the databases in personal/research group websites [59,60] or in on-line repositories, such as The Community Resource for Archiving Wireless Data At Dartmouth (CRAWDAD) platform [61,62,63,64,65], UCI Machine Learning Repository [66,67,68,69,70], or Zenodo [71,72]. To spread their availability, data-descriptor papers can be published [73] or databases can be referenced/included as Supplementary Materials of a particular research work [71].

As commonly done in machine learning, the evaluation of an indoor positioning system using a wide variety of datasets represents its general accuracy better since many evaluation contexts are considered. In addition, the availability of datasets allows comprehensive comparative studies that provides researchers with a better overview of all works related to, for instance, an indoor positioning technology. Unfortunately, there is not a well-established consensus about how to collect (strategies to generate the dataset) and provide this data (file format) yet.

## 3. The IPIN 2017 Competition

The Evaluating Ambient Assisted Living initiative (EvAAL) aims at establishing benchmarks and evaluation metrics for comparing Ambient Assisted Living solutions and it has organised international competitions on indoor localization and indoor activity recognition since 2011.

In 2017, EvAAL members applied their experience to the organization of IPIN 2017 Competition, which was held during the IPIN Conference (16–21 September 2017, Sapporo, Japan). The competition was composed of four tracks:

- Track 1: Smartphone-based (on-site)
- Track 2: Pedestrian Dead Reckoning Positioning (on-site)
- Track 3: Smartphone-based (off-site)
- Track 4: PDR for warehouse picking (off-site)

This paper focuses on the organization, results and lessons learned in the 2017 IPIN competition’s Smartphone-based (off-site) track, which might be considered as a continuation of the Smartphone-based track from the previous IPIN competition. The following subsections will describe the main aspects of this database off-site competition.

### 3.1. The Off-Site Track Rules

The main features of the 2017 IPIN Competition - Smartphone-based (off-site) track were:

1. An off-site competition approach where the organizers provided the required data for calibration, validation and evaluation. It was forbidden for competitors to survey the evaluation scenarios by themselves. This constraint guaranteed that all competitors had the same data for participating in the competition. All competitors were notified about the existence of public-access databases that were collected for the previous 2016 IPIN Competition [74].
2. The data were provided as logfiles recorded by a dedicated Android application (*GetSensorData v2*, which is available at [75] and which was first used in [32]). A logfile contains sensors’ measurements and landmark labels recorded while the actor followed a continuous trajectory.
3. A natural movement is assumed in most captured data. Some special movements were also considered: turns, moving backward/laterally at certain points and changing floors through stairs. The user speed was approximately constant while recording the data with stops at some positions. It is important to note that the data were collected by a human, and not a controlled constant speed device, which makes the data closer to a natural movement.
4. None of the collected dataset included artificial phone holding. The smartphone was either stable in front of actor’s face or chest, which is a typical position for reading or typing on the phone, or with the arm downwards while holding the phone with a hand. In only very few cases, the phone was near the actor’s ear simulating a phone call. This unpredicted variability is closer to reality.
5. Each logfile covered a trajectory in one of the evaluation buildings and it was recorded by an actor using a smartphone. The provided data are diverse because they consist of multiple trajectories taken at three different buildings. A total of eight people participated in the database creation and 10 devices were used.
6. The scenarios remained unaltered during the collection process. Only one of them changed, because BLE beacons had been previously deployed in a small area for testing purposes. The information about the location of those beacons was provided to competitors.

With respect to the 2016 IPIN off-site competition [74], the competition organizers introduced only a few changes. Most features were kept in order to create a stable data format and maintain the natural way of capturing the information, and also to encourage previous competitors to participate again, year after year, as the learning curve for them to compete is low. The few changes added in this current 2017 edition were introduced with the goal of increasing the interest for the competition itself, such as adding an additional RF technology (BLE), and providing an additional natural phone motion for a more challenging positioning problem. Those changes are summarized as follows:

1. Bluetooth Low Energy data were available.
2. Position of BLE beacons and a few Wi-Fi APs was provided.
3. In a few cases, the actor simulated a phone call while data were being captured, to add a natural motion.
4. More raw-data per building were provided.

The competition environment comprised a total of three buildings: CAR (CSIC Arganda, Madrid, Spain), UJIUB (Universitat Jaume I, Castellón, Spain) and UJITI (Universitat Jaume I, Castellón, Spain), all of which were also part of the 2016 IPIN Competition.

#### Description of Datasets

Raw geo-referenced data were provided as a set of logfiles for training and validation purposes, which corresponds to a stream of sensor data registered sequentially. Each line in the file corresponds to a register, a single sensor reading, stored as plain text. The register fields are separated by semicolons and contain the values provided by the sensor, the timestamps and a header that identifies the type of sensor. The register named POSI does not correspond to any sensor and it provides the ground truth location provided by the users. The sampling rate of each kind of sensor depends on the Android device and operating system version.

Three types of datasets were available to competitors: training, validation and test. There was no substantial difference between them regarding the sensor’s registers. Only the training and validation logfiles contained the POSI registers with the geo-referenced positions of the reference points. The position was provided as latitude and longitude (WGS84 format) and the identifiers for floor and building.

All datasets were simultaneously published on April 2017, i.e., five months before the deadline that the competitors had for submitting their location estimation results. Table 1 shows the information about the collected logfiles in each scenario.

Similarly, Table 2 shows the information about the collected data in the IPIN 2016 competition for comparative purposes. First, the 2017 IPIN Competition provided validation data, which allowed competitors to test their systems with geo-referenced data. In 2016, the validation strategy relied on the competitor and specific data for this purpose was not provided. Second, all datasets were simultaneously published in April 2017, instead of the two stage release (training and test) done in 2016. Third, the number of buildings was reduced to three because the organizers performed a more exhaustive data collection in each building and due to the available time for creating, checking and publishing all datasets. Despite this reduction, in 2017, the organizers provided ≈40% more data with respect to the 2016 edition. Considering only the three buildings, in 2017, the organizers provided an increase of ≈134% in accumulated distance, of ≈83% in reference positions, and of ≈170% in accumulated time in comparison to the 2016 competition.

Once the datasets were actually used in the competition, some issues were found:

- Regarding the ground truth data, which were provided by POSI lines in training and validation logfiles, some competitors suggested to have more reference data and shorten the period between two consecutive POSI entries in the logfiles. However, the data collection, which included marking each reference point, was not an easy task.
- The organizers included a tricky transition between floors in one of the logfiles. This transition (stairs area) was not included in the training and validation logfiles and was considered a good way to test the robustness of competing IPS when facing unexpected events.
- The floor penalty was applied whenever a competing system misidentified the floor. However, in floor transitions (stairs areas), a system may provide an estimation very close to the evaluation point and yet be prone to miss the floor. Some competitors indicated that future competitions should take into account these special cases. The competition organizers are considering to integrate a more realistic error distance metric [76] to calculate the positioning error in next editions.

The competition organizers appreciate the honest feedback from competitors and will work on balancing the competitors suggestions and competition challenges.

### 3.2. Submission of Results and Evaluation

After processing the evaluation logfiles (test subsets), participants could perform up to three different submissions of indoor location estimates. The estimates had to be provided at a pace of 0.5 s, starting from the first timestamp available in each logfile. The estimations had to adhere to the following format: timestamp, latitude, longitude, floor, and building.

Each submission was evaluated considering the estimates provided for the seven evaluation logfiles. The base positioning error was defined as the geometric distance between the two-dimensional (latitude and longitude) real position as recorded by competition organizers and the estimated position provided by the competing IPS. This base error is commonly referred as two-dimensional positioning error or X-Y error. Furthermore, we took into account floor and building mis-identifications during the trajectories. In this case, 15 and 50 m penalties were added to the geometric error if the IPS had not correctly estimated the floor and building, respectively. Thus, the positioning error is defined in this paper as the two-dimensional positioning error plus the penalties terms. The final metric used for the competition results was the third quartile (75% percentile) of this joint positioning error.

Of the three alternative sets of estimations submitted by the competing teams to the tests, only the one with the best results was considered for the ranking of competitors.

### 3.3. The Competing Teams

This section highlights the main features of the competing teams and references their works in the IPIN 2017 proceedings:

- The UMinho team: Adriano Moreira, Maria João Nicolau, António Costa, Filipe Meneses. University of Minho and Centro de Computação Gráfica, Guimarães, Portugal.
- The AraraDS team: Tomás Lungenstrass, Joaquín Farina, Juan Pablo Morales. AraraDS, Santiago, Chile.
- The Yai team: Wei-Chung Lu, Wen-Chen Lu, Ho-Ti Cheng, Shi-Shen Yang, Shih-Hau Fang, Ying-Ren Chien and Yu Tsao. Yuan Ze University, National Ilan University, Academia Sinica Research Center for Information Technology Innovation, Taiwan.
- The HFTS team: Stefan Knauth. Stuttgart University of Applied Sciences, Stuttgart, Germany [77].

#### 3.3.1. UMinho Team

The approach used by the UMinho team on the 2017 edition of the competition is a refinement of the solution used in the previous year competition [78]. The core of the positioning estimation solution is a plain Wi-Fi fingerprinting estimation algorithm based on the k-nearest neighbours classifier. The optimum use of this base algorithm in the context of the IPIN 2017 competition required, however, a set of additional data manipulation and estimation processes. A brief description of these additional techniques is provided next. One of the challenges of using fingerprinting with the provided datasets is that a radio map has to be constructed, which requires the estimation of the positions where the samples were collected. This is a consequence of the time offset between the POSI records and the time instant when fingerprints were collected (refer to Moreira et al. [78] for the used approach). Compared to the previous edition, the problem of building the radio map extended to the new BLE fingerprints. The approach of the UMinho team was to experiment with two solutions: (i) build one single radio map by merging Wi-Fi and BLE fingerprints into a single vector (considering BLE access points as Wi-Fi access points); and (ii) build independent radio maps, one for Wi-Fi and one for BLE, and then use one or the other to estimate the position associated to each testing Wi-Fi or BLE fingerprint, respectively. This solution aimed at exploring the added information provided by the BLE samples, where available. The second enhancement was related to the use of barometric pressure data to help in estimating the correct floor. A barometric pressure profile is defined as a smoothed version of the temporal variation of the measured atmospheric pressure along the route. Pressure profiles are useful to detect changes in the floor along the route, but cannot be used to estimate the absolute floor. Therefore, the absolute floor estimations provided by Wi-Fi fingerprinting was used to automatically fit the pressure profile. While in 2016 these data were always used when they were available in the testing datasets, the solution of 2017 used the barometric pressure data only after verifying the correlation between the pressure profile (the time series representing the estimated floor along the time) and the floor estimated through the Wi-Fi fingerprinting estimator. When a good correlation was observed, the pressure profile was used to better define the time instant of transition between floors; otherwise, only the Wi-Fi estimations were used as they proved to be more reliable. Information about the mobility profile of the users (being in motion or standing still) can be used to improve the performance of positioning methods based on fingerprinting. Among other aspects, fingerprints collected while the user is not moving can be merged together to generate a less noisy fingerprint and, potentially, improve the position accuracy. The UMinho approach to create a mobility profile, for each route, is based on processing accelerometers’ data and detect steps [78]. Additionally, visual inspection of the accelerometers’ data was used to estimate the initial smartphone calibration procedure. While information about this process has not been provided to the competitors, the patterns observed in the accelerometers’ data suggests that such procedure has been used at the beginning of most of the routes. In this year’s competition, the process to estimate the mobility profile was improved, and then used to merge all the Wi-Fi fingerprints collected while the user was detected as standing still.

#### 3.3.2. AraraDS Team

AraraIPS is Arara’s proprietary indoor positioning technology. This startup has rapidly grown during its four years of existence to become the largest Wi-Fi network administrator in Chile. Arara is engaged in developing advanced knowledge solutions and producing high-quality technology to address modern business and industry challenges. AraraIPS has been one of the central issues in their research agenda for the last year. In spite of being still in an early development stage it is already a functional and promising indoor positioning system.

AraraIPS’ approach to indoor positioning has four distinctive characteristics: it is based on a cartographic paradigm (fingerprinting), it uses a discretization of the predicted floor/building, it is measurement-agnostic (i.e., its abstract formulation is not specific to any kind of signal or measurement such as Wi-Fi, magnetic field, BLE, etc.), and it exploits measurement history. In the following paragraphs, these features will be shown to get a better understanding of how AraraIPS works.

Fingerprinting has become a relevant technique in indoor positioning approaches. Traditional positioning methods are generally based upon one such principle as trilateration or triangulation, in which sufficient geometric information (with respect to reference landmarks) singles out the position of the object to be located. These methods are based on underlying hypotheses (e.g., clear line of sight between object and reference landmark is needed to establish a functional one-to-one relation between distance and signal intensity) which usually do not hold in the dynamic, cluttered context of indoor spaces. Even though cartographic approaches still need to take care of changing environment, they can adapt to obstruction and other conditions that naturally occur in indoor spaces and thus avoid the pitfalls of traditional positioning techniques. From Arara’s point of view, this is a crucial property for a successful positioning system.

**Figure 1:**
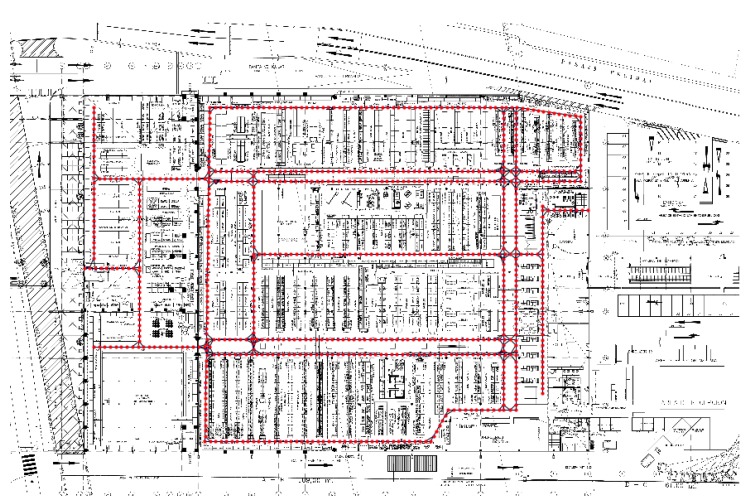
Sample graph on a hardware store map.

Arara’s system also relies on the discretization of the underlying indoor space. In practice, this means that a graph is built from the map of a venue, in which nodes are possible locations where the tracked device can be found and edges connect neighboring nodes. This effectively turns the positioning problem into a classification one, in which the prediction is one of finitely many possible locations. It has the further advantage of ruling out inaccessible locations (e.g., walls) and thus not complicating the prediction task unnecessarily. An example of a generated graph is shown in Figure 1.

Fingerprinting also has the additional advantage of not needing any a priori knowledge about the nature of the measured quantities (provided that they are effectively vector fields in the indoor space). Arara’s system was expected to be as scalable and flexible as possible, and this was taken as a feature of the model. This is not a property that provides improved performance, but a desirable commercial feature for AraraIPS to be able to adapt fully and simply to varying operating conditions. Therefore, the system uses a very general probabilistic model for measurements taken at a given location, which is common to all mapped quantities.

To make the system scalable, which is a fundamental concern, it does not rely on the installation and maintenance of additional hardware. The Arara’s team considers that the available information (Wi-Fi, magnetic field, and possibly BLE) under typical operating conditions in most buildings is not discriminative enough by itself to yield a sufficiently precise prediction. Hence, the solution proposed (and the final ingredient in the system) is to enrich the information available to the prediction module by taking into account the history of measurements, exploiting the fact that measurements taken close in time will be strongly correlated. Thus, it is expected to narrow in the prediction on the true position, with greater and greater confidence as time goes by. The mathematical formulation of this is enclosed in what the Arara’s team called “random walk model”. The output of the algorithm is thus a probability distribution over all possible nodes.

For the competition, the fingerprinting method was adapted to use the available data. The predictions submitted are almost solely based on Wi-Fi information (GNSS is used outside buildings and PDR was “cosmetically” used for prediction interpolation in one submission with no actual improvement in results). This was mostly because the system is currently under development and reliable PDR techniques have not yet been implemented/developed. One of the submissions of Arara’s team included an experimental feature using future information to improve present predictions with worse, inconsistent results. More work is still needed to look deeper into it to understand what is actually going on.

#### 3.3.3. YAI Team

An increasing growth of indoor localization solutions has been witnessed during the past few years. Although many different positioning approaches exist, the YAI team uses the traditional fingerprinting approach to address indoor positioning in the IPIN competition. Positioning was divided into three main stages parts:

1. Determination of the correct building;
2. determination of the correct floor; and
3. estimation of the latitude and longitude coordinates.

First, the YAI team uses the GPS data to determine the correct building. Due to the buildings distribution, this stage should not require any other advanced method in order to determine the correct building.

Second, the Wi-Fi data were used to determine the floor differences. Wi-Fi fingerprints were labelled with the floor numbers. Then, the corresponding access point and its RSS value are used to calculate the Euclidean Distance between the training fingerprints and the testing fingerprints. The minimum distance in the RSSI space provided the predicted floor number. Pressure data was used to revise the final prediction of floor.

Third, data from Wi-Fi, Accelerometer and Magnetometer were used to determine the coordinates (longitude and latitude). This algorithm consists of two differentiated fine-grained positioning systems:

- if the distance between the two consecutive points is less than 5 m, the accelerometer and magnetometer data are used to predict the trajectory of the walking person; and
- if the distance between the two consecutive points is more than 5 m, Wi-Fi Fingerprinting approach is used to determine the coordinates.

The members of the YAI team found that using accelerometer and magnetometer data provided accurate results but the mean error increased remarkably as the distance between two points became larger. Thus, the threshold value was set to 5 m in order to switch to the fingerprinting-based positioning system.

In the Wi-Fi fingerprinting based approach, the YAI team labelled the Wi-Fi data with coordinates. Then, the corresponding access point and its RSS value were used to calculate the Euclidean Distance between the training point and the testing point. Finally, the minimum distance provided the predicted coordinates.

The competing system introduced by the YAI team was tested on the validation logfiles. The building identification was 100%, whereas the floor identification was higher than 95%. The mean error of all three building is 4.412 m for building CAR, 4.532 m for building UJITI and 4.829 m for building UJIUB. The results using the validation data were in line with the results provided in previous competitions for the three buildings.

#### 3.3.4. HFTS Team

The HFTS team combined PDR/Wi-Fi algorithms [77] and employed GNSS, Wi-Fi, accelerometer, compass, and gyroscope data. In their system, step detection is performed by peak detection of the accelerometer data, and compass and gyroscope are used for heading estimation. Drift compensation and step length estimation is performed by a particle filter using the information of floor plans to detect the most likely path (see also [79,80]). Floor detection is based on position and received signal strength indicator (RSSI) evaluation. RSSI positioning is performed using the scalar product correlation fingerprinting algorithm [81].

Heading estimation is performed by combining gyroscope and magnetic information: the gyroscope is able to detect heading changes quite accurately on a short timescale, but will drift in the long-term. Therefore, the algorithm stabilizes the gyroscope heading with the compass heading. Compass heading is subject to strong local magnetic perturbations but shows no drift on a long-term scale. The estimated heading is calculated by summing gyroscope heading changes immediately, but relaxing back to magnetic heading with a configurable constant.

The particle filter contains a constant number of particles. Besides the position, a particle state also comprises individual step length and heading offset values. The filter is updated each time a step is detected: All particles are moved according to the estimated heading, individually modified by the particle specific offsets. Each time a particle collides with, for example, a wall, it is replaced by a new one. For collision detection, the provided floor plans are used. New particles are seeded at the position of an existing particle, but with an own step length and heading offset value.

A particular feature of the algorithm is its backtracking capability. By recursively tracking back the history of a particle, a continuous track can be estimated for each individual particle, allowing a posteriori elimination of failed particles. This leads to a significant accuracy increase for cases where the position information is not needed in real time.

The HFTS 2017 competition algorithm includes fusion of PDR and Wi-Fi results: To keep the concept of continuous particle movement, i.e., no sudden displacement of particle positions, the Wi-Fi information is used to adjust the particle movement parameters which are heading and step length. This is described by the following equations:

(1) $$\begin{array}{c} l^{\prime }=\left(1-a_{step}\right)l+a_{step}l\frac{d_{Wi-Fi}}{d_{PDR}} \end{array}$$

(2) $$\begin{array}{c} \Theta^{\prime }=\Theta +a_{head}\cdot \arccos\frac{d_{PDR}\cdot d_{Wi-Fi}}{d_{PDR}\;d_{Wi-Fi}} \end{array}$$

Starting at the position $R(t_{1})$ of a past time $t_{1}$, the vector $d_{PDR}$ to the current position $R{\left(t_{2}\right)}_{PDR}$ is compared with the vector $d_{Wi-Fi}$ connecting from $R(t_{1})$ to the position $R{\left(t_{2}\right)}_{Wi-Fi}$, which is obtained by Wi-Fi. From the obtained distances $d_{Wi-Fi}$ and $d_{PDR}$ a corrected step length $l^{\prime }$ Equation (1) is calculated; from the angle between the vectors, a corrected heading $\Theta^{\prime }$ Equation (2) is derived. To balance between PDR and Wi-Fi positions, coupling factors or gains $a_{step}$ and $a_{head}$ are introduced for the step- and the heading correction. Using values in the interval [0,1] the effect of RSSI on the PDR position can be adjusted. While for $a_{step}$ typically small values below 0.1 were used, the angle gain has been useful set to values of 0.5.

On stairs, the step length changes considerably and is dependent on the stairs stride. It is typically about half of the normal step length. This is taken into account by marking stair areas with a dedicated color in the floor maps. The collision check also includes a stair check based on the map marking. The particle filter then adjusts the step length accordingly to a predefined value, for the corresponding particles. The stair information is also used in the handling of floor change processing: Floor changes may only occur when passing stairs. At the moment when a floor change is detected, all particles not situated on a marked stair area are removed from the particle filter. This increases the convergence of the particle filter. The scheme could also be used to handle elevators or escalators.

The particle filter may also get empty i.e., all particles are discarded. This may for example happen when the number of particles is too small, the heading estimation has a high error or the floor plan is not correct, i.e., people can walk through walls. A possible approach to solve the issue could be to seed new particles at random positions for example around the known Wi-Fi position. However, the reseeding of new particles at new positions cuts the link to the particles history, thereby disabling the particular backtracking feature of the algorithm.

The chosen solution is to suspend the collision detection for a short time in cases where all particles would collide. The confinement of the particle cloud is then performed by the Wi-Fi position: Particles for which the distance to the Wi-Fi position exceeds a configurable value are discarded.

In summary, the algorithm exhibits the following particular features:

- PDR Offset cancellation is not only map-based but also Wi-Fi based: The particle movement process of the particle filter includes a Wi-Fi based heading offset and step length correction. Depending on parameter setting, this may lead to ignoring Wi-Fi, mixing Wi-Fi and inertial sensor information or completely adjust to Wi-Fi positions.
- The particle filter uses Wi-Fi results as a bound to remove outliers: By keeping an estimated Wi-Fi position, particles exceeding a configurable distance from the Wi-Fi position are removed from the particle filter, thus keeping the particle cloud confined to a certain radius.
- The PDR algorithm considers floor areas on which a level change may occur, e.g., stairs and elevators. These areas are marked manually on the floor map. This allows step length adjustment for stairs and, in combination with Wi-Fi floor change detection, particle filter “flushing”, e.g., removal of particles which are not on a floor change area.
- In cases where the particle filter loose all particles, the map requirement is withdrawn, e.g., particles may cross walls. They are still confined to some extend by the Wi-Fi position.

## 4. Results

This section presents the track 3 IPIN 2017 competition results and, after that, those results are compared with the results of other competitions.

### 4.1. Competition Results

**Figure 2:**
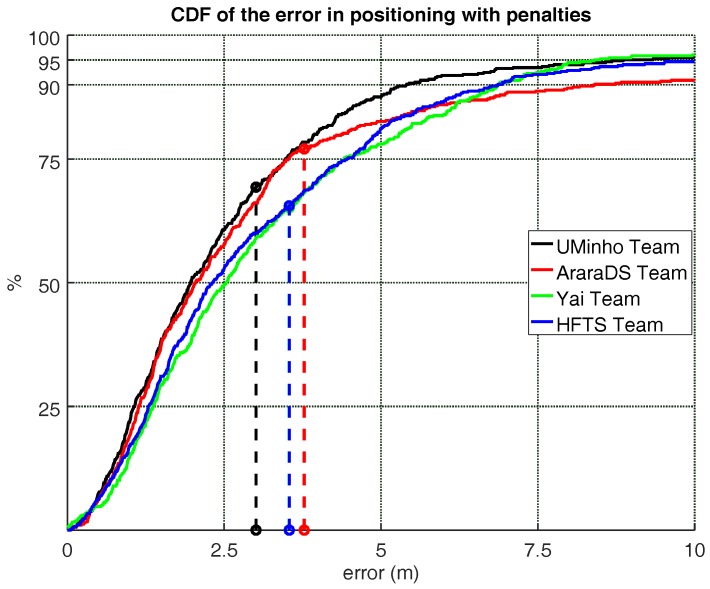
Competition Results in CDF (Cumulative Distribution Function) representation for the seven logfiles as a whole (CDF represented by a solid lines for each team). Alternative metric based on the mean positioning error (applying floor penalties) is represented by vertical dashed lines.

The competition results for the total 505 evaluation points are shown in Table 3 and Figure 2, with special emphasis on the third quartile of the positioning errors with penalties (the competition score). All results correspond to the best set of results (the one reporting the lowest score) among the three sets submitted by all competitors.

The winner of this edition was the UMinho team (Score: 3.48 m) followed closely by AraraDS team (Score: 3.53 m). The difference between their scores was just 5 cm, which demonstrates the quality of both competing systems regarding the competition metric. The Yai and HFTS teams were third and fourth in the competition rank, respectively. The CDF shown in Figure 2 ratifies the competition principal rank. The values presented in Table 3 are the error values that Figure 2 shows associated to 75 percentile for each team. Figure 2 also presents with vertical dashed lines the mean positioning error (with penalties included).

### 4.2. Detailed Results

Although the ranks provided in the IPIN competition correspond to the third quartile of the positioning error, we consider that more details are needed to understand why a comprehensive evaluation of indoor positioning systems (including different scenarios and contexts) is useful.

**Figure 3:**
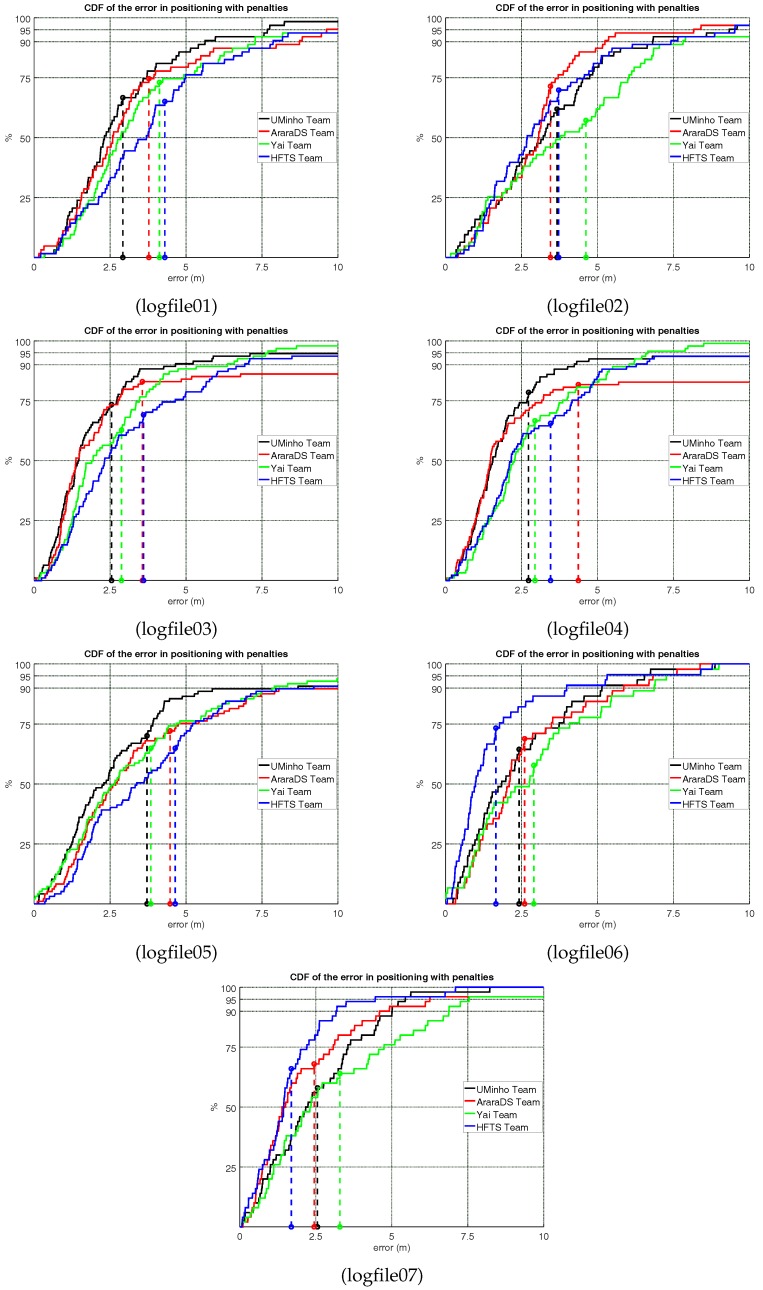
Competition Results—Cumulative Distribution Function of the positioning error plus the mean positioning error (vertical dashed lines) in each logfile.

The detailed extended evaluation results are shown in Table 4 and Figure 3, where results on each of the logfiles are shown (third quartile, mean positioning error, two-dimensional error (or horizontal error) and floor detection rate). The table also includes two aggregated results in the top and bottom lines: *All logfiles* and *Average*. The *All logfiles* metric corresponds to the mean positioning error or the floor detection rate for the 505 evaluation points as a whole. The *Average* metric line, considers the average of the mean results shown for the seven logfiles. In all these metrics, all logfiles correspond to the best set of results out of the three files allowed to be presented by the competitors.

The results provided by the two aggregation alternatives (*all logfiles* and *average*) slightly differ from the competition metric since they are based on the average of the positioning error and hit detection rate instead of the third quartile. In addition, both aggregation alternatives are different to each other as the numerical results in the table reflects. This difference is because the former aggregation alternative averages all reference points with equal weight and the later corresponds to the average of the averaged accuracy of each logfile. In this second approach (*average*) the weight among all points is different as not all logfiles contain the same amount of reference points, so that shorter experiments have more weight.

In view of these alternative results, and comparing them to the third quartile official metric, the competition organizers consider that the main metric used in the competition represents the general accuracy of the competing systems, but the extended information is also useful for developers and community to understand how each system works on each considered scenario and context.

Although the UMinho team provided the best score and aggregated results, it is worth noting that the AraraDS team provided the best results in logfile 2; the Yai team provided the highest floor detection in the evaluation logfiles collected at the UJIUB building (logfiles 3, 4 and 5); and the HFTS provided the lowest mean positioning error in the logfiles collected at the UJITI building (logfiles 6 and 7). This fact demonstrates that comprehensive evaluation procedures are useful, not only to obtain a winning system but also to show the complexity of developing an indoor positioning system that provides the best results in all contexts and scenarios.

According to the individual CDF plots, it can bee seen that the CDFs for logfiles 1, 3, 4 and 5 (Figure 3) resemble the CDF for the positioning results considering all logfiles (Figure 2).

Logfiles 1 and 2 correspond to CAR building. The CDFs show that the UMinho and AraraDS teams are the bests for logfile 1, and the AraraDS and HFTS teams are the best ones for logfile 2. Although both evaluation logfiles were collected almost simultaneously by two independent actors following a similar trajectory, logfile 2 was collected by means of a “new” device (not used in training and validation) which might have negatively affected a few competing systems (especially for the UMinho and Yai teams).

Logfiles 3, 4 and 5 correspond to the UJIUB building and the three CDFs are similar. It seems that the results provided in logfile 5 are slightly worse than in the other two logfiles collected in the same building (logfiles 3 and 4).

Logfiles 6 and 7 correspond to two independent trajectories in the UJITI building. The CDFs show that the HFTS team was the best team in this building (with a mean positioning error lower than 2 m in both cases).

The individual results and CDFs show that the results are not homogeneous and they depend on the scenario and context. The UMinho team won the competition, but the AraraDS and HFTS were the best in three individual logfiles using the competition metric (the third quartile). The accuracy does not only depend on the scenario, but also on the evaluation context: particular disturbances present in the environment, device used for positioning or, even, actor interaction as the results of logfile 5 show.

Finally, Table 5 introduces a comparison of the results provided by the winners of the Track 3 in the 2016 and 2017 editions. Although the main score reported in 2017 is 2 m lower than in 2016, one challenging building was not considered in the 2017 edition, therefore a direct comparison of both scores should not be done. To overcome this issue, the comparison can be done building by building or by computing the average error considering all the logfiles belonging to the CAR, UJITI and UJIUB buildings. For the CAR building, the results are worse than in 2016, even though BLE tags were introduced in this building. For the UJIUB, the results are better (considering the positioning error and floor detection rate). For the UJITI building, the results in both editions are similar (the difference is just 24 cm). The average error is similar in both editions (2.73 vs. 2.96), but the deviation is lower this year, which indicate that the average values are more representative for the three scenarios in 2017. Considering the CAR, UJITI and UJIUB buildings, the 2016 and 2017 competitions were similarly challenging for the competing teams.

It is worth noticing that the most challenging scenario in the 2016 edition was the UAH building according to the winner’s results; this fact could make us reconsider the re-inclusion of this building or the inclusion of challenging huge scenarios such as shopping malls in the evaluation process. The main problem of including this kind of scenarios is the extraordinary time needed to obtain a dense enough surveying.

At first sight, the organisers have detected that the results are more coherent in 2017 than in 2016 edition. Two teams provided very competitive results with a difference of just 5 cm in the competition metric, but the other two teams also provided good accuracy. The difference between the winner and the last ranked team is ≈1 m in 2017. In 2016, the difference of the winner with respect to the runner-up team was much higher (≈1.5 m), and one competing team provided a very large positioning error (higher than 40 m). It seems that providing a large set of training and validation data has benefited all competitors at the expense of a comprehensive data log procedure from the organization team.

Competitors in the current competition that also participated in previous editions were able to evaluate their systems over the time and track the implemented modifications. Moreover, having the historical data is useful for the indoor research community because a real comparative study of working systems is shown in real-life scenarios.

### 4.3. The IPIN Competition Track 3 and Other Competitions

The results provided in the Microsoft (MS-IPSN) and IPIN Competitions are shown in Table 6, whereas Table 7 shows their evaluation features: number of tracks/trajectories, total evaluation length (accumulated distance between evaluation points), number of reference/evaluation/control points, and the total evaluation duration.

The context must be considered when comparing the results provided by the competition winners. The technological solution that won the MS-ISPN 2017 competition provided the lowest mean positioning error in the table (3 cm) and it was the best solution in its competition track. Although the positioning error is very low, it cannot be directly compared to the winners of the other competitions since the competitions do not share the same requirements. The winner of the MS-ISPN 2017 competition was based on LIDAR, which is not allowed in smartphone and PDR based applications and competition tracks. In addition, we cannot state that the winner of the IPIN 2017 Competition Track 3 is better than the winner of the IPIN 2017 Track 1 (Smartphone-based competitions), because the evaluation scenarios and rules were different in both tracks. In particular, the evaluation scenario of the IPIN Tracks 1 and 2 was very challenging in 2017, and the competitors of IPIN 2017 Tracks 1 and 2 had to provide the position estimations in real-time.

Another important difference among the competitions is how the tracks (or categories) are defined. In the IPIN competition, the tracks are device oriented (smartphone or PDR mainly). In addition, the IPIN competition provides different tracks for on-site and off-site competitions, even if the same base device is used. In the MS-ISPN competition, a black-box evaluation is adopted and the systems are evaluated with independence of the technology employed for localization. In 2014 and 2015, the MS-ISPN competition provided two competition categories according to the infrastructure required for positioning (infrastructure-free or infrastructure-based). In 2016 and 2017, the MS-ISPN categories were 2D and 3D positioning. In general, the existing competitions cover different aspects of categorising the indoor positioning in order to consider the wide diversity of indoor positioning systems. Thus, the importance of having all those diverse initiatives to compare indoor positioning systems.

The appropriate context is also relevant for the features shown in Table 6. Although the competitions share a common goal, the evaluation of indoor positioning systems, the target evaluated systems and contexts are different.

First, the Microsoft competition aimed at a real-time evaluation of working competing systems, where the evaluator stood for a few seconds at pre-defined test points. Competitors had a strict time slot for their system evaluation (15–20 min) and they were able to sense the environment and deploy any hardware the day before. Due to time and logistic constraints, the number of evaluation points was between 15 and 20 and the evaluation scenario comprised only a few rooms and corridors. Furthermore, the categories established are not oriented to a particular technology. Systems using visible light, ultrasounds, IMU or Radio-frequency based systems, among others have competed under the same category. Smartphone based systems have also participated in this competition, being a Wi-Fi fingerprinting-based system the winner of the infrastructure-free category in 2014.

Second, the IPIN Competition (Tracks 1 and 2) also aimed at a real-time evaluation in multi-floor environments, where an actor followed a trajectory defined by a sequence of test points. The evaluation trajectory covered the IPIN conference venue and its access was not restricted (it was not a closed test environment). Since the evaluation path was long and covered different floors (in all editions) and buildings (only in 2015 edition), the evaluation of competitors was done in parallel. In IPIN Tracks 1 and 2, hardware deployment in the evaluation area was forbidden. Due to time constraints, the number of evaluation points was about 60 and the evaluation length trajectory was about 500–600 m. Track 1 has been devoted to smartphone-based positioning, whereas foot-mounted devices have been used in Track 2 since 2015.

Third, the IPIN Competition (Track 3) aimed at the off-site evaluation of smartphone-based solutions in complex multi-floor environments. Since the competition was run off-line and off-site, there was not strict time restrictions to meet. In the on-line competitions, the organizers have usually had one day to evaluate all competing teams during the conference. With an off-site competition, the competition can be done in advance and the results can be presented during the conference. The competition timings allows the collection of longer evaluation trajectories—with an accumulated length of ≈4.4 km and ≈3.2 km in 2016 and 2017, respectively—that include many evaluation points—more than 500 in both editions.

In real-time on-site competitions, it might not feasible to evaluate each competitor in a extremely long trajectory or in a very large scenario. On the one hand, the timing and logistic restrictions might be a severe issue. In current on-site competitions, more than 20 teams are evaluated the same day. In addition, competitors can deploy (potentially expensive) hardware in some competitions (e.g., the Microsoft Competition allows infrastructure-based systems), whose costs might be prohibitive in very large scenarios. On the other hand, the probability of having a wide variety of unexpected errors might increase during the evaluation in long evaluation trajectories, such as application hangs, battery drain, or actor’s fatigue, among many others.

The off-site competitions, such as IPIN Track 3, offer the possibility of evaluating the positioning accuracy in long tracks with a high number of reference points. This evaluation procedure assures that all competing teams have at their disposal the same data and information about the environment at calibration and evaluation stages. Moreover, diversity is introduced in the evaluation paths since data are gathered in different buildings, by different people and with different devices (smartphones in this case). However, having a very large evaluation path with diversity is not a synonym of being a better evaluation procedure. The evaluation procedure used in the off-site competition cannot take into consideration the ability of the competing teams to successfully deploy their localization infrastructure in the environment for positioning purposes. In addition, the off-site competition cannot take into consideration the ability of the competing teams to perform an optimal survey of the environment and successfully calibrate an indoor positioning system for the evaluation area. Finally, the real-time component is not present in the off-site competitions, where the competitors have the opportunity of post-processing the position estimated before its final submission. In addition, the competing systems in on-site competitions must be robust because if they fail during the evaluation window, they might not have another chance to compete.

We consider that the strategies followed in real-time on-site competitions are suitable for evaluating indoor positioning systems. Off-site competitions can be considered a complementary evaluation strategy, where a more comprehensive evaluation (in terms of positioning error calculated over a large evaluation path with embedded diversity) can be done. However, the “comprehensive” evaluation of the positioning error in off-site competitions is done at the expense of not considering some hidden evaluation aspects, for example, the availability of the indoor positioning system, ability to successfully adapt or customize the competing system to a particular scenario in a few hours, and ability of optimally surveying the environment, among other features.

## 5. Conclusions

In this paper, we have shown the results obtained by the competitors in Track 3 of the IPIN 2017 competition, regarding off-site evaluation of smartphone-based solutions. Results have been compared with previous (2016) competition and with other competitions.

### 5.1. Competitors’ Feedback and Lessons Learned

The competitors provided to the organizing team many suggestions to improve the competition, being the most representative ones:

- Provide more detailed data without gaps (e.g., without skipping multiple floors or rooms).
- Try to minimize unexpected events like missing stairs areas in training and validation log files.
- Code the floor transitions with a special floor code (e.g., 3.5 for transitions between the third and fourth floors) to minimise the unfair large positioning errors near areas close to floor transitions.

After a positive discussion with competitors, the organizers detected two major concerns about the competition.

- *Registration of datasets*. The registration of data in the logfiles for the competition is done in a continuous way with minor stops. Some competitors, especially those that mainly rely on fingerprinting, felt uncomfortable with this procedure because they prefer a static registration, or at least a very slow continuous motion (0.2 m/s), in order to have more stable Wi-Fi fingerprints and more samples. They also would like a dense survey with many points, and no testing experiments outside of the surveyed area. We understand this point of view, but we also consider the current relevance of the generation of fingerprinting solutions from incomplete or low sampled scenarios, surveyed by actors in motion (the way that crowd-sourced fingerprints are registered by persons at malls, for example). Next editions could include some sites with a high density of points taken at static positions, just to observe how some systems can benefit from it, but we consider that we should keep the core of the current registration approach.
- *Metric fairness*. Regarding the metric used in the IPIN competition, which is based on the third quartile plus some penalties generated by floor or building identification mistakes, some competitors suggested that the weights for those penalties were too large (for example, the 15 m penalty for a wrong floor estimation). The usage of those penalties emphasizes that a positioning service must pay high importance to guaranteeing a correct floor indication. For some applications, a wrong floor indication can be very adverse for the location service provided. For example, a mistake in a floor number can make the user to lose valuable time when navigating in a mall from one section to another, and, most importantly, could lower the user interest in that service since it is perceived as misleading. The 15 m penalty is even a small penalty considering the time needed to correct our route from a failure due to a floor mistake. The competition organizers are considering in further editions to determine the positioning error using a more realistic distance, which computes the walkable distance between the estimated and real position.

To improve the future editions of the off-site smartphone-based competition, the aspects analysed in this paper must be reinforced, maintained, promoted or changed. The main goal of the competition is to provide a forum and a evaluation procedure that embrace all competitors while keeping challenging competition conditions that mimic realistic and diverse environments.

### 5.2. General Conclusions

The general conclusions reached after the competition are as follows.

- *Databases are important for comprehensive comparisons*. The creation of different databases, in different buildings, with different actors, different smartphones, and with a high degree of diversity is, in general, the only way we can analyse the performance of systems with enough detail. We have seen from our analysis that some teams created algorithms whose behavior was better in a certain environment, or even for test occurrences, than others. Our feeling is that the typical evaluation procedure for IPS found in the literature, where a team selects a particular building and compares its method with a ground truth or with another approach, is prone to excessive fine-tuning of algorithms, and insufficient to conclude that the proposed positioning approach is robust and accurate enough for real-life uncontrolled scenarios. Competitions are a good way to collect experimental data of diverse sites for the creation of banks of databases against with to compare.
- *Competition motivates competitors from Academia*. Research involved in non-commercial applications does not commonly have the pressure of providing the best accurate working systems in every possible scenario. Once the details of their base system have been published (using their own experimental set-up in most of cases), the resources are dedicated to explore novel aspects and features of indoor localization. However, an annual competition that involves challenging participants from academia or industry forces every competitor to be highly competitive and to develop accurate working systems, ad not just prototypes. This fact boosts their motivation to improve and refine their systems to levels that are not usually considered in traditional R & D works.
- *Competition integrates Academia and Industry*. Competitions can be seen as showrooms where both worlds, Academia and Industry, show their developments, advances and interesting findings. Although the main aim of a competition is to compete and win, synergies between the competitors can be consolidated after the competition. In fact, informal contacts between competitors were detected after presenting the competition results. Creating a forum, to discuss about indoor positioning and establish new contacts, was one of the competition pillars.
- *Competitions must be diverse*. Although this paper presents the results of experiences of the 2017 off-site smartphone-based competition, there are other interesting competitions and evaluation initiatives available which consider different base positioning technologies (not smartphone-based such as PDR, UWB, and Ultrasound), real-time evaluation, joint off-line and on-line evaluation, among many other evaluation features. We consider that diversity in competitions is important, because it allows a huge indoor positioning community to be represented.
- *Competitions need to cover multiple scenarios and contexts*. The results of Track 3 from IPIN 2017 Competition show an interesting finding: none of the competing systems provided the best accuracy (positioning error, X-Y error and floor hit detection rate) in all the evaluation logfiles. This finding demonstrates that the accuracy of a positioning system depends on the scenario and context. Therefore, to have a comprehensive evaluation, as many as possible scenarios and contexts have to been considered in the evaluation set-up, thus avoiding winners that provide high accuracy for specific settings but perform poorly under other scenarios and contexts. However, considering multiple scenarios and contexts might not be feasible in on-site competitions, because the time constraints are very tight. Even for off-site competitions, the effort of considering multiple scenarios and contexts is huge, because the time required for planning the data collection, gathering the raw data and performing post-processing is considerable.
- *Competitions need continuity*. Although all competitions are important, the ones with continuity are particularly relevant. Continuity is an essential feature in order to track the competition evolution and to keep the motivation of competitors. Annual competitions, such as the IPIN and MS competitions, enable competitors to participate each year. The teams from the University of Minho and the Technical University of Stuttgart have participated in all three editions of this competition and they have received valuable feedback to refine their systems.

Finally, one of the most important findings of this paper is that the evaluation procedure of indoor positioning systems should be diverse and include different evaluation contexts and scenarios. Finding a golden method encompassing in all scenarios and contexts was not easy in the 2017 IPIN competition (Track 3, Smartphone-based off-site) according to the detailed results presented in this paper: each competing system stands out in a particular context or scenario in the 2017 competition. Although the UMinho team provided the best competition scores, the other competing systems also provided interesting partial results. Although diversity in evaluation is desirable, it is not possible under some circumstances. It is worth mentioning that a trade-off between diversity and time constraints has to be met in on-site competitions, which is less strict in off-site competitions. In general, all competitions have had a positive impact on the evaluation of indoor positioning systems and the positioning research field.

## Figures and Tables

**Table 1:** Resume of the logfiles provided to 2017 IPIN competitors.

Scenario	Subset	Logfiles	Total Length (m)	Num. Ref. Points	Total Duration (s)
CAR	Train	9	≈3720	450	9551
	Validation	3	≈1865	225	4512
	Test	2	≈1180	126	2620
UJITI	Train	4	≈3795	424	4105
	Validation	2	≈410	59	874
	Test	2	≈590	95	1134
UJIUB	Train	12	≈3425	702	4755
	Validation	4	≈1450	332	2544
	Test	3	≈1450	284	2764
	total	38	≈17,890	2697	32,859

**Table 2:** Resume of the logfiles provided to 2016 IPIN competitors.

Scenario	Subset	Logfiles	Total Length (m)	Num. Ref. Points	Total Duration (s)
CAR	Train	4	≈2180	254	4295
	Test	2	≈1085	152	2447
UJITI	Train	2	≈1640	561	1724
	Test	2	≈740	121	673
UJIUB	Train	5	≈1615	294	2286
	Test	1	≈375	91	730
UAH	Train	6	≈3035	320	5603
	Test	4	≈2200	214	4755
	total	26	≈12,860	2007	22,513
	total *	16	≈7630	1473	12,155

total * considers only CAR, UJITI and UJIUB buildings.

**Table 3:** Competition Results—Third Quartile of the positioning error with penalties (in meters) on the 505 test points.

	UMinho Team	AraraDS Team	Yai Team	HFTS Team
Score (m)	3.48	3.53	4.41	4.45

**Table 4:** Extended Competition Results—3rd quartile (3rd Q), Mean Positioning error (MPE), Two-dimensional error (X-Y) and Floor detection rate (Flr) on each logfile and aggregated metrics

	**UMinho Team**				**AraraDS Team**			
	**3rd Q (m)**	**MPE (m)**	**X-Y (m)**	**Flr (%)**	**3rd Q (m)**	**MPE (m)**	**X-Y (m)**	**Flr (%)**
All Logfiles	3.48	3.00	2.44	96.24	3.53	3.74	2.67	92.87
Logfile 01	3.59	3.06	3.06	100	3.78	3.72	3.72	100
Logfile 02	4.51	3.73	3.73	100	3.60	3.46	3.46	100
Logfile 03	2.72	2.51	1.87	95.74	2.76	4.04	2.12	87.23
Logfile 04	2.67	2.74	1.77	93.55	3.17	4.14	1.72	83.87
Logfile 05	3.91	3.70	2.31	90.72	4.74	4.47	3.23	91.75
Logfile 06	3.73	2.46	2.46	100	3.48	2.65	2.65	100
Logfile 07	3.56	2.55	2.55	100	3.07	2.43	2.13	98
Average	3.53	2.96	2.53	97.14	3.52	3.56	2.72	94.41
	**Yai Team**				**HFTS Team**			
	**3rd Q (m)**	**MPE (m)**	**X-Y (m)**	**Flr (%)**	**3rd Q (m)**	**MPE (m)**	**X-Y (m)**	**Flr (%)**
All Logfiles	4.41	3.51	3.36	99.01	4.45	3.52	2.89	95.84
Logfile 01	4.24	4.08	4.08	100	4.94	4.19	4.19	100
Logfile 02	6.00	4.66	4.66	100	4.32	3.83	3.83	100
Logfile 03	3.47	2.86	2.54	97.87	4.65	3.64	2.68	93.62
Logfile 04	3.85	2.97	2.81	98.92	4.16	3.43	2.47	93.55
Logfile 05	4.68	3.86	3.55	97.94	5.22	4.63	3.24	90.72
Logfile 06	4.09	2.95	2.95	100	1.77	1.67	1.67	100
Logfile 07	4.75	3.39	3.39	100	2.21	1.71	1.71	100
Average	4.44	3.54	3.43	99.25	3.90	3.30	2.83	96.84

**Table 5:** Average results of 2016 & 2017 IPIN Competition winners in the different evaluation scenarios.

	2016 Winner (Score: 5.85)		2017 Winner (Score: 3.85)	
	MPE (m)	Flr (%)	MPE (m)	Flr (%)
Average logfiles CAR	1.98 ± 0.35	100 ± 0	3.4 ± 0.47	100 ±0
Average logfiles UJIUB	5.16 ± 0	89.01 ± 0	2.98 ± 0.63	93.34 ± 2.52
Average logfiles UJITI	2.27 ± 0.33	100 ± 0	2.51 ± 0.06	100 ± 0
Average logfiles UAH	10.37 ± 6.55	93.18 ± 8	-	-
Average (all logfiles except UAH)	2.73 ± 1.39	97.8 ± 4.91	2.96 ± 0.55	97.14 ± 3.85

**Table 6:** Indoor Competitions—Winner’s results.

Competicion	Track	Winner’s Accuracy (m)	Metric
MS-ISPN 2014	infrastructure-based	0.72	MPE
MS-ISPN 2014	infrastructure-free	1.76	MPE
MS-ISPN 2015	infrastructure-based	0.31	MPE
MS-ISPN 2015	infrastructure-free	0.2	MPE
IPIN 2015	Smartphone (on-site)	6.6	3rd Quartile
IPIN 2015	PDR	2.4	3rd Quartile
IPIN 2015	Smartphone (off-site)	8.3	3rd Quartile
MS-ISPN 2016	2D Positioning	1.2	MPE
MS-ISPN 2016	3D Positioning	0.16	MPE
IPIN 2016	Smartphone (on-site)	5.4	3rd Quartile
IPIN 2016	PDR	1.5	3rd Quartile
IPIN 2016	Smartphone (off-site)	5.8	3rd Quartile
IPIN 2016	Robotics	0.1	3rd Quartile
MS-ISPN 2017	2D Positioning	2.2	MPE
MS-ISPN 2017	3D Positioning	0.03	MPE
IPIN 2017	Smartphone (on-site)	8.8	3rd Quartile
IPIN 2017	PDR	2.04	3rd Quartile
IPIN 2017	Smartphone (off-site)	3.48	3rd Quartile

**Table 7:** Indoor Competitions—Features of the evaluation trajectories.

Competition	Trajectories	Length (m)	Ref. Points	Duration (s)
MS-ISPN 2014	1	N/A	20	<1200
MS-ISPN 2015	1	162	20	<900
IPIN 2015 Tracks 1 & 2	1	645	62	852–981
MS-ISPN 2016	1	81	15	<900
IPIN 2016 Tracks 1 & 2	1	674	56	718–1129
IPIN 2016 Track 3	9	4398	578	8605
IPIN 2016 Track 3 *	5	2198	364	3850
MS-ISPN 2017	1	91	20	N/A
IPIN 2017 Track 1 & 2	1	530	58	667–809
IPIN 2017 Track 3	7	3220	505	6518

* Considering only the buildings present in the IPIN 2017 Track 3.

## Supplementary Materials

The following are available online at: http://indoorloc.uji.es/ipin2017track3/files/logfiles2017.zip.
